# Breaking barriers in cancer diagnosis: unveiling the 4Ms of biosensors

**DOI:** 10.1039/d4ra08212e

**Published:** 2025-03-17

**Authors:** Sachin Gupta, Vijay Mishra, Alaa A. A. Aljabali, Aqel Albutti, Rajeev Kanday, Mohamed El-Tanani, Yachana Mishra

**Affiliations:** a Department of Robotics and Control Engineering, School of Electronics and Electrical Engineering, Lovely Professional University Phagwara Punjab-144411 India; b School of Pharmaceutical Sciences, Lovely Professional University Phagwara Punjab-144411 India; c Department of Pharmaceutics & Pharmaceutical Technology, Yarmouk University Irbid Jordan alaaj@yu.edu.jo; d Department of Basic Health Sciences, College of Applied Medical Sciences, Qassim University Buraydah 51452 Saudi Arabia; e School of Computer Science and Engineering, Lovely Professional University Phagwara Punjab-144411 India; f Ras Al Khaimah Medical and Health Sciences University Ras Al Khaimah United Arab Emirates; g School of Bioengineering and Biosciences, Lovely Professional University Phagwara Punjab-144411 India yachanamishra@gmail.com

## Abstract

Cancer, an insidious affliction, continues to exact a heavy toll on humanity, necessitating early detection and nuanced comprehension of its intricacies for effective treatment. Recent strides in micro and nanoscale electronic chip fabrication have revolutionized biosensor technology, offering promising avenues for biomedical and telemedicine applications. Micro Electromechanical System (MEMS)-based integrated circuits (ICs) represent a paradigm shift in detecting chemical and biomolecular interactions pertinent to cancer diagnosis, supplanting conventional methodologies. Despite the wealth of research on biosensors, a cohesive framework integrating Material, Mechanism, Modeling, and Measurement (4M) dimensions is often lacking. This review aims to synthesize these dimensions, exploring recent breakthroughs in biosensor design and development. Categorized based on electromechanical integration, material selection, and fabrication processes, these biosensors bridge crucial knowledge gaps within the research community. A comparative analysis of sensing methods in point-of-care (PoC) technology provides insights into their practicality and efficacy. Moreover, we critically evaluate biosensor limitations, pivotal in addressing challenges hindering their global commercialization.

## Abstract 2

Cancer, an insidious affliction, continues to exact a heavy toll on humanity, necessitating early detection and nuanced comprehension of its intricacies for effective treatment.

## Graphical abstract

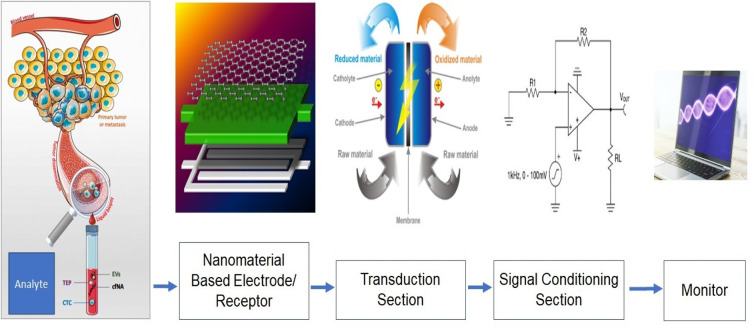

## 1. Introduction

A major problem in contemporary medicine, cancer affects millions of individuals globally. Globally, GLOBOCAN estimates that 2.2 million instances of cancer, including many forms of cancer, have been diagnosed as being caused by infections. Accurate diagnosis is essential in the battle against cancer because of its worldwide relevance. This necessitates the creation of novel techniques that can distinguish between various cancer forms and offer individualized care to every patient. The timely identification and diagnosis of cancer cells are essential for formulating successful treatment plans.^1,2^

The generally recognized gold standard for cancer diagnosis is the enzyme-linked immunosorbent test (ELISA), which identifies protein biomarkers unique to malignancy. Additionally, molecular techniques based on genomics and proteomics, such as radioimmunoassay (RIA), immunohistochemistry (IHC), and polymerase chain reaction (PCR), are utilized in the detection of cancer. Furthermore, a variety of clinical instruments are made considerable use of, including computed tomography (CT), endoscopy, sonography, MRI, PET, and X-rays. Though the technologies and procedures listed above are effective, the majority of them are costly, time-consuming, intrusive, and only available in certain hospitals' laboratories. It may be difficult to definitively identify cancer tumors smaller than millimeters, especially when using imaging techniques. Similar issues and challenges arise when identifying early-stage cancer tumors using invasive techniques like biopsy.^3^

The high specificity and sensitivity of PCR procedures, which enable the identification of genomic material with as few as 10 oligomers, are among the benefits. RT-PCR analysis requires expensive equipment, a lengthy analysis, and precise control as limitations in diagnosis to prevent false positives caused by contamination. LAMP is a gene amplification method that enables quick and accurate DNA detection. A LAMP's low detection threshold, simple operation, and rapid amplification (it can generate 109 copies of DNA per h) all work in its favor. The widely recognized and often applied microscope agglutination test (MAT). Even though MAT is the gold standard for serological testing, it nevertheless demands a high degree of technical proficiency. Furthermore, ELISA has potential since it can be conducted in a greater variety of laboratories at a lower cost than the MAT test. Immunoglobulin M (IgM) and other biomarkers generated in the body may be measured using ELISA to diagnose an infection brought on by a disease. Biosensor devices are one of the most promising alternatives for the diagnosis or therapeutic monitoring of major diseases such as cancer. They are anticipated to provide fast and accurate biomedical analysis with small sample sizes and no pretreatment. Early diagnosis and monitoring of pathological conditions, particularly cancer disease, may significantly improve prognosis and survival rates through the analysis of molecular biomarkers using biosensor platforms. This would reduce the burden of disease, support social development, and increase access to healthcare for people worldwide.^4–7^

The rapid advancement of technology, exemplified by Lab-on-Chip (LoC) systems incorporating Micro Electromechanical Systems (MEMS) and the fabrication of nano-chips using biocompatible materials, has revolutionized Point-of-Care (PoC) telemedicine in the field of biomedicine.^8^

The pressing need for early disease detection and continuous monitoring of disease progression and regression cannot be overstated. Chip-based devices have emerged as vital tools capable of precisely measuring biological processes and transmitting this critical information to receivers, enabling a proactive approach by medical practitioners. In alignment with the vision of medical experts and the scientific community, there is a growing demand for the development of ready-to-use electrochemical chambers at the nanoscale, designed with a plug-and-play approach and built for biocompatibility. These chambers aim to monitor specific signals of interest for early detection through the PoC approach.^9^ These cutting-edge developments have enabled clinical tools and procedures to be seamlessly embedded into micro and nanoscale chips, facilitated by bio-fabrication and state-of-the-art technology.^10–14^

The effectiveness of a portable LoC system with an external sample extraction procedure in identifying cervical cancer from biopsy samples is demonstrated by Wormald *et al.* The apparatus uses loop-mediated isothermal amplification (LAMP) tests in conjunction with ion-sensitive field-effect transistor (ISFET) sensors to amplify human telomerase reverse transcriptase (hTERT) mRNA and HPV DNA. These markers were chosen due to their low to nonexistent expression in normal cervical tissue but high levels of expression in cervical cancer cells. The limit of detection for hTERT was reached with an analytical sensitivity of 102 for the molecular targets, which resolved down to a single copy per reaction for the mRNA markers. All malignant tissues and no benign tissues had hTERT mRNA identified; the pilot results suggest that the LoC might be widely used for cervical cancer screening in resource-constrained environments.^15^

The effectiveness of the LoC technology in combination with variant-specific isothermal amplification techniques for the identification and separation of wild-type (WT) and mutant (MT) copies of the ESR1 gene is demonstrated by Alexandrou *et al.* Hormone-resistant malignancies frequently increase the risk of metastatic illness, which raises death rates, particularly in places with limited access to healthcare and poor economic levels. Compared to other common industry-similar technologies like qPCR and sequencing, the LoC system that is being demonstrated here is smaller and less expensive. The suggested LoC platform offers mutational tracking of circulating tumor DNA in liquid biopsies to support patient stratification and metastatic surveillance, and it may be utilized in a point-of-care diagnostic context for breast cancer (BC).^16^

A groundbreaking method developed in Sunny *et al.*'s study, tele-cytology combined with the risk stratification model, can be a priceless PoC tool for early detection/screening in oral cancer.^17^ In a three-armed parallel randomized pilot research, Alacevich *et al.* assigned recently referred patients with financial toxicity and cancer to either normal care plus instructional materials or individual, group-certified telehealth financial counseling (1 : 1 : 1). The financial toxicity (COST) and Telehealth Usability Questionnaire (TUQ) scores were used to evaluate the feasibility of recruitment, randomization, retention, baseline, and post-intervention comprehensive scores. This pilot research evaluated the viability of a PoC intervention to link adult patients experiencing financial toxicity due to cancer to financial counseling provided by telehealth.^18^

A typical biosensor consists of the following components: (a) bioreceptors that bind specifically to the analyte; (b) an interface architecture where a particular biological event occurs and results in a signal picked up by (c) the transducer element; the transducer signal, which can be anything from the current generated at an electrode to the in-coupling angle of a laser beam, is amplified by a detector circuit using the appropriate reference and sent for processing by, (d) computer software to be converted to a meaningful physical parameter describing the process being investigated; finally, the resultant quantity must be presented through (e) an interface to the human operator.^19^ Electrochemical biosensors are classified as (i) potentiometric, (ii) amperometric, (iii) impedimetric, (iv) conductometric, and (v) voltammetric based on the transduction principle. The potential difference that builds up between the reference and indicator electrodes during the recognition process in an electrochemical cell when very little or no current passes through the electrode is the basis for the potentiometric transduction operation.^20^

It is important to note that the potential of biosensors in the field of cancer diagnosis and monitoring is still being explored and refined. The complexity of cancer and the intricacies of its biomarkers present unique challenges that require meticulous research and development efforts. Separating and enriching the circulating tumor cells (CTCs) in advance using CTC capture methods, followed by the introduction of the enriched CTC into electrochemical chambers, the addition of substrate, and aptamer/antibody microbeads (which have catalytic properties) is a major process for cancer detection. The microbeads can catalyze the reaction, induce color change, or generate gas; by monitoring the color change, pressure change, or pressure-driven distance, the CTC can be quantified. Well-designed channels, columns, and chambers would allow for effective cell separation. It also accomplished high-throughput detection, allowing for the quick analysis of hundreds of samples. The microfluidic chip's low cost, ease of use, and little reagent usage are its most crucial features.^21^

**Fig. 1:**
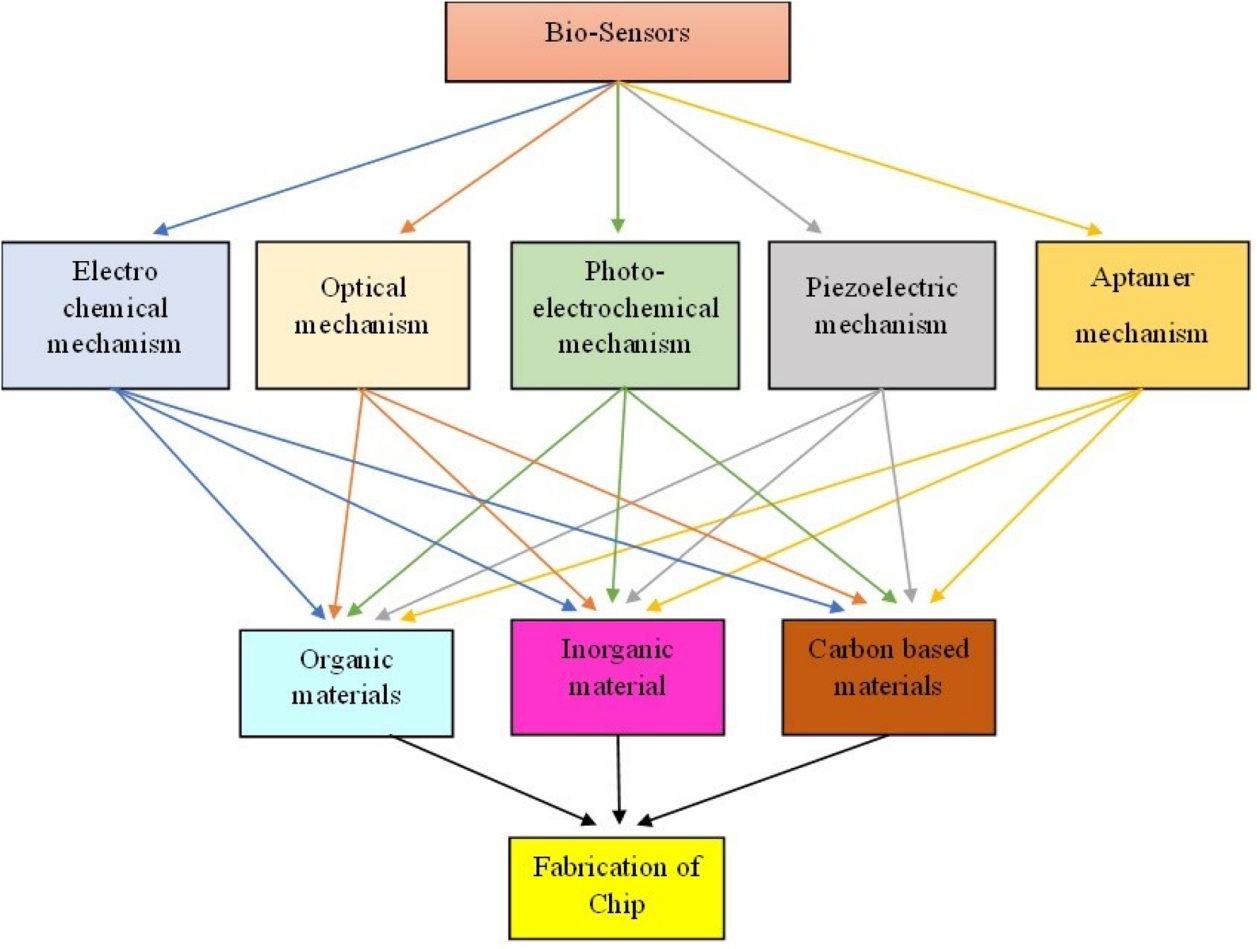
Materials and mechanisms involved in cancer-based biosensors.

In this comprehensive review, we delve into the recent breakthroughs in biosensors and the associated processes. Notably, we explore cutting-edge research concerning the amalgamation of materialistic structures presented across distinct sections. Fig. 1 serves as an illustrative depiction of the materials and methodologies employed in the development of biosensors engineered for the detection and identification of various cancer biomarkers.

## 2. Architecture and functioning of biosensors for cancer detection

**Fig. 2:**
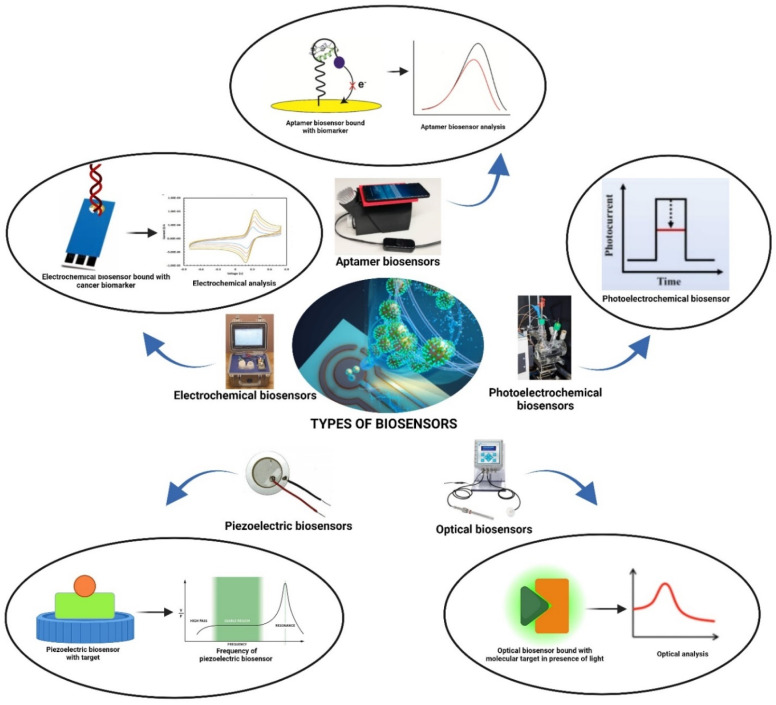
Different types of biosensors used for cancer detection.

The biosensing field has experienced exponential growth, leading to the displacement of traditional methodologies for detecting an array of compositions, including DNA, proteins, and cancer-related biomarkers.^22–25^ Biosensors adhere to a standardized architecture, which is categorized into different transduction principles encompassing electrochemical, optical, photoelectrochemical, piezoelectric, and aptamer sensors (Fig. 2). These diverse sensor types leverage a wide array of compatible materials, facilitating the precise detection of cancer biomarkers.^26–34^ These advanced biosensor assemblies are meticulously designed in a specific sequence, intricately tuned to the precise detection of the biomarker in question. This strategic alignment of materials, mechanisms, and methodologies represents a significant advancement over conventional techniques, enhancing sensitivity and accuracy in detecting critical cancer biomarkers.^4^

### 2.1 Architecture of biosensors

While various cutting-edge technologies are continuously being created in laboratory settings, the procedures and methods for diagnosing cancer that are used in clinical practice have not evolved significantly over the past few decades. There are two distinct steps to the conventional diagnostic process. An invasive tissue biopsy of the afflicted area is first carried out. Medical imaging, such as computed tomography (CT), magnetic resonance imaging (MRI), or positron emission tomography (PET), frequently comes next. Although these techniques have received a great deal of validation and are therefore commonly used, they have some inherent drawbacks, including being intrusive, requiring laborious procedures, and being expensive. Although blood tests have made it possible to screen people more quickly and diagnose illnesses earlier, they are still quite intrusive, particularly for those who are weak. Although commercially available ELISA kits have a considerable test duration, they offer high sensitivity.^35^ Reagents used in radioimmunoassay are unstable and will eventually degrade, and measurement repeatability is not good. Despite the ease of use, high sensitivity, and accuracy of fluorescence immunoassay, the tedious labeling procedure makes quantitative analysis difficult. High-information biological materials and biomolecular signal systems can be obtained by Raman spectroscopy. However, for high-sensitivity quantitative and qualitative analysis, conventional Raman signal spectra are too faint to be often employed. In this sense, biosensors provide significant benefits, including quick reaction times and simpler methods.^36,37^

The development of biosensors dedicated to cancer detection stands as a prominent and focused area of research for scientists and research communities. The multifaceted nature of biosensor engineering necessitates a synthesis of knowledge spanning diverse fields. Biosensors represent electromechanical devices with a hierarchical structure, commencing with a bioreceptor at the apex, followed by a transduction segment, and culminating in an analyte composition. These devices harness electrochemical or optical principles to amplify biological activity and convert it into discernible electrical signals through a myriad of physiochemical reactions.^38,39^ To address the challenges unique to the measurement of circulating proteins clinical samples, Nasrollahpour *et al.* developed an enzyme-free electrochemical biosensor based on electrosynthesized biocompatible WO_3_/poly glutamic acid nano-biocomposites. Apart from its environmentally friendly manufacturing process, the produced nano-biocomposite exhibited exceptional performance in untreated biological samples, including high protein content due to the low amine group propensity of both components. With a broad linear response between 1 ng mL^−1^ and 1 fg mL^−1^, the HER-2 concentration was detected with a nanobiosensor as low as 1 fg mL^−1^. Meanwhile, the procedure demonstrated optimal repeatability, stability, and specificity for HER-2 protein detection in untreated blood samples from BC patients.^40^

It demonstrates how to use a surface plasmon resonance-based optical fiber biosensor that is both affordable and portable for early BC detection. The HER-2 protein is highly expressed in BC, which is frequently linked with more aggressive tumour development and a worse prognosis; thus, a HER-2 aptamer may be utilized to identify the existence of HER-2 positive BC. The antigen–antibody binding approach is used to determine the presence of the HER-2 protein. Notably, the biosensor's reaction time was shown to be as quick as 5 s. The suggested biosensor provides a route to early BC prevention and shows promise for early detection of HER-2 protein in first cancer serum.^41^

Micro-electromechanical systems (MEMS) technology serves as a versatile platform, facilitating the customization of chips wherein various modules are intricately integrated to execute specific functions, such as the detection, injection, implantation, and tissue repair within the human body. These biosensors encompass multiple sections meticulously designed to capture proportional or linear signals in response to target analyte concentrations.^42–44^

Advancements in chip lithography techniques have paved the way for the precise formation of electrodes, crucial components of biosensors, using micro- and nano-scale sputtering methods. Various nanomaterials, including carbon fiber, gold nanoparticles (AuNPs), silver nanoparticles (AgNPs), and paper-based materials, are harnessed in the creation of electrode assemblies.^45^ In line with contemporary lithography methods, biosensor-based electrodes can be meticulously molded into diverse sizes, shapes, and fusion-based aspect ratios, thereby enhancing their capacity to detect signals.^46^ In one study, lithographic micro-electromechanical systems were used to create a comb of microchannel and immunosensor based on long-period fiber grating for the real-time and label-free detection of particular antigens. A microchannel long-period fiber grating (CM-LPFG) comb was used to connect the propagating core and cladding modes. The CM-LPFG-based immunosensor was made of an optical fiber sandwiched between a microchannel structure created using photoresist stacking techniques and a long-period structure. To detect specific protein antigen from the extracted protein mixtures of the cancer cell lines, a particular antibody against protein antigen was attached to an optical fiber surface and created a real-time resonance effect. Based on the diversification of a transmission loss, it was discovered that the CM-LPFG-based immunosensor for the real-time detection of label-free protein antigen is practical and sensitive, and it fulfils specific immuno-sensing objectives for lab-on-fiber (LoF) technology.^47^

Recent research underscores the pivotal role of material fusion in enhancing the detection of specific biomolecules. Fusion-based electrodes exhibit remarkable properties, offering linear responses across electrical, magnetic, and optical domains. These electrodes outshine conventional laboratory-based diagnostic techniques such as biopsy, chromatography, or light-based spectroscopy by several orders of magnitude.^48^ In summary, biosensors are poised to revolutionize the approach to the prognosis and detection of cancer-causing agents, introducing unparalleled levels of precision and efficiency.

In the scope of this review, we explore various categories of biosensors, dissecting their operational principles and assessing their success rates in identifying cancer symptoms. The comparative analysis of diverse research endeavors provides a comprehensive analytical perspective on the progress within the biosensing sector.^49,50^

### 2.2 Functioning of biosensor for identification of biomarkers

When it comes to designing biosensors, nanomaterials play a pivotal role in crafting the electrode assembly. Where the analyte and substrate converge to produce a selective signal specific to the target analyte. This critical juncture is where the transduction principle comes into play, serving as the sole mechanism responsible for extracting essential information from the generated signal. Within this framework, chemical reactions give rise to a diverse array of signals, each accompanied by various types of labels. The transduction principle serves as the discerning agent within this hypothetical cluster of signals, isolating the specific signal indicative of the target analyte. In the context of cancer-related molecule detection, various transduction processes are explored and discussed in detail.

#### 2.2.1 Electrochemical transduction

The electrochemical-based transduction principle stands as one of the most widely adopted methods for detecting biological markers, particularly in the realm of enzyme-based sensors. In the nanoscale assembly of biosensors, this transduction principle emerges as a highly favored tool for efficient and time-effective target analyte detection. Its robustness allows for the handling of complex serum solutions while minimizing interference from other molecules.^51^ Notably, electrochemical-based biosensors demonstrate a remarkable resilience to external biological and environmental conditions, as documented in the literature. Recent research highlights the primary goal of design assembly: the detection of key biomarkers with attributes such as high specificity, linearity, accuracy, a low limit of detection, and a serene response to the target analyte within dynamic environments.^52^ Ongoing progress in the development of electrochemical-based biosensors also accentuates structural engineering efforts to create miniaturized and universally adaptable biomarker detectors. The electrochemical transduction mechanism hinges on physiochemical reactions between biomolecules. When the biosensor electrode assembly encounters the serum or relevant fluid sample, a chemical reaction takes place between the selected analyte and the target analyte.^53^ This reaction gives rise to an electrical signal containing crucial information. This signal is characterized by various physical aspects, such as signal amplitude, frequency, phase shift, impedance variation, current or capacitance, magnetic response, and material inductance. The signal conditioning module can process this single signal into multiple physical modes, effectively deciphering valuable insights about the target analyte. Smartly designed biosensors enable this multiplexity of models, aligning with the concept of LoC, which holds the potential to supplant conventional methods.^54^ The electrochemical-based transduction biosensor detects the movement of electroactive molecules within a complex serum. The corresponding signal is measured to determine concentrations, guided by the generic principle of altering electrical parameters like capacitance, resistance, and potential differences. With advancements in fabrication technology, the reference and measured signals can be directly compared to the chip itself.^55^ Comparative analyses, spanning various physiochemical forms such as potential differences, changes in resistance, electron flow, and phase differences, are employed to quantify the signal corresponding to the actual concentration of cancer-related molecules.

Numerous recent studies emphasize the calibration of these biosensors for re-use, tailored to specific patent situations. The ability to fine-tune biosensors provides medical practitioners with a valuable tool for continuously monitoring dynamic conditions within tissues. This approach not only improves the PoC concept but also does so at a cost-effective rate.^56^ A profound understanding of the electrochemical transduction principle's mechanisms for signal processing is imperative. The electrical signal processing unit scrutinizes the signal across a spectrum of transduction mechanisms, encompassing voltammetry, amperometry, potentiometry, and electrochemical impedance variations. The transition of the measured signal from the electrode to the signal conditioning module within the electrode assembly relies on careful control and manipulation of impedance matching between modules. This represents a pivotal task in the creation of multilayer-based hybrid biosensors. Throughout the electrochemical process, the strength of the physiochemical signal fluctuates within a range of 1 μA to 100 nA. Effective signal transmission hinges on the creation of a well-structured biosensor that incorporates a fusion of nanomaterials, allowing for the formation of compatible impedance matching between modular components, such as gel or nanowire strings. Consistent impedance matching ensures that the spectrum of signals can be readily observed, aiding in making informed decisions regarding cancer stage management.^57^

Nanomaterials exhibit diverse properties and can be categorized based on a range of attributes, including mechanical strength, temperature stability, thermal conductivity, inertness, electrical responsiveness, and physiochemical behavior. Within the realm of polymeric organic nanomaterials (ONMs), several subcategories have emerged as key players in the fabrication of cancer-based biosensing modules. These include polymer nanocomposites, molecularly imprinted polymers, dendrimers, hyperbranched polymers, nanostructured hydrogels, nanofilms derived from polymers, and covalent–organic frameworks. ONMs possess distinctive electrochemical properties, characterized by their remarkable inertness, rapid response kinetics, and robust physical integrity, making them ideal for immobilizing electrons within electrode assemblies. In contemporary research, metallic nanostructures such as nanowires, nanocages, and nanoshells composed of silver, platinum, and gold have garnered significant attention due to their exceptional sensitivity in detecting target analytes.^58^

Furthermore, carbon-based nanomaterials (CNMs) are emerging as prominent contenders in biosensing applications. Carbon allotropes have found utility in tissue and structural engineering, driven by their high surface-to-mass ratio. Notably, their lightweight nature, cost-effectiveness, and potential for reusability have raised considerable interest. Advances in tissue engineering and vascular structure design have enabled the shaping of carbon into various forms, including carbon nanotubes (CNTs), carbon dots (CDs), graphene-based materials, nanodiamonds, and fullerenes. The fusion of these carbon-based materials holds promise for the design of cancer-based biosensors. To delve deeper into the electrochemical transduction process facilitated by these materials and its role in enhancing biomarker specificity with heightened sensitivity and superior detection limits, please refer to Table 1.

**Table 1:** Response of biosensors based on electrochemical transduction mechanism

Material	Nanomaterial base	Target analyte	Size (nm)	Range of detection	Limit of detection	Key features	Ref.
Organic	Graphene/Ag/Au/polyaniline	PSA	42–99	1 pM to 10 mM	<1.02 pM	Several organic NMs have proven effective in detecting tumor markers, such as prostate-specific antigen (PSA), human carcinoembryonic antigen (CEA), alpha-fetoprotein (AFP), human chorionic gonadotropin (hCG), human epidermal growth factor receptor-2 (HER-2), cancer antigen 125 (CA125), cancer antigen 15-3 (CA15-3, MUC1), and cancer antigen 19-9 (CA19-9) with great sensitivity and selectivity	59
	Nano MIPs acrylamide monomer/PVC	P53	80–125	1 nM to 1 mM	1 nm	As the suggested sensor has a surprisingly high sensitivity spanning a concentration range of 0–10 000 ppm, as well as a high quality factor (QF) and figure of merit, it might be an important component of a viable platform for detecting low quantities of various heavy metal ions in freshwater	60
	PNC (Phc-PSy)	CA-125	25–49	0.012–125 U mL^−1^	10–4 U mL^−1^	Diagnostic procedures and developments achieved with LCs are gaining increased sensitivity due to their streamlined strategy and multiplexed detection	61
	Au/MoMA/LoPr	NSE	24–81	1.01 pM–10.01 nM	0.77 pM	Organic semiconductor nanoparticles (OSNs) have various benefits in biosensing applications, including strong fluorescence emission, variable emission wavelength, and good biocompatibility. Furthermore, by applying alternative design methodologies, a diverse spectrum of functional probes that suit distinct biosensing needs have been produced	62
Inorganic	Graphene–palladium–platinum	PSA	15–60	1–300 μm	0.3 μM	The research investigates how organic framework emitters are incorporated into biosensor systems to improve sensitivity and selectivity for a variety of analytes. The authors compare organic framework emitters to standard inorganic emitters, highlighting benefits such as improved biocompatibility, simplicity of functionalization, and the possibility of multiplexed detection	63
	Gold–graphene NCg	CEA	30–80	6–100 μm	0.5 μm	This article introduces the principles and mechanisms of electrochemiluminescence (ECL) biosensors for biomarker determination, as well as recent advances in ECL biosensors on key aspects such as new ECL reagents and materials, new biological recognition elements, and emerging construction biointerfacial strategies, with illustrative examples and a critical eye on pitfalls. It also discusses the challenges and perspectives of ECL biosensors in health analysis	64
	Gold NCgs-nanotubes	SCCA	20–50	0.3–0.6 μm	0.1 μm	Nanomaterial-based biosensor systems provide high conductivity, a wide surface area, and great electron transferability to immobilized biomolecules	65
	AuPtAg-ANCs	PSA	50–150	0.17 ng mL^−1^	0.05–50 ng mL^−1^	Biosensors are useful for improving therapy success and patient survival since they need minimum invasion. Carbon elements, on the other hand, have a wide range of characteristics at the nanoscale, making them ideal for biosensor manufacturing. As a result, carbon nanomaterials such as graphene and carbon nanotubes are employed as elite nanomaterials in healthcare-related biosensors	66
Carbon allotropes	Graphene oxide nanosheet	AFP	30–95	0.01–300 ng mL^−1^	6.4 pg L^−1^	Electrochemical (bio)sensors made from nanodiamonds have been used to detect a wide range of species of interest, including organic chemicals, medicines, hormones, glucose, heavy metals, nitroaromatic explosives, and pesticides	67
	Au NPs/NH_2_-GS	AFP	17–90	10–198 fg mL^−1^	3.3 fg mL^−1^	The surface area, porous network, hybridization, functionalization, synthesis route, combination of dimensionalities, purity levels, and mechanisms underlying carbon nanomaterial interactions all have an impact on biosensor technology applications in bioanalytical chemistry	68
	ND-Di hexadecyl phosphate film	CA19-9	09–30	0.299–108 μm	54.5 nM	Various functionalized CNMs have a high capability for improving cell targeting of anti-cancer medicines. Because of their thermal capabilities, they have been widely utilized in cancer photothermal and photodynamic treatment, which is supplemented with laser irradiation and CNMs. The CNMs can also pass the blood–brain barrier, allowing them to treat a variety of brain ailments, including neurodegenerative diseases, by eliminating amyloid fibrils	69
	CNT-amended graphite electrode	TP53	10–65	0.05–1 nM	18 μm	The biosensor displayed great storage stability of 63 days, as well as high accuracy in detecting ANXA2 in blood samples from LC patients, as verified by the enzyme-linked immunosorbent assay method	70

Contemporary research endeavors are dedicated to the detection of potential cancer biomarkers, including exosomes, through the utilization of hybrid and meticulously structured biosensors integrated with electrochemical sensing platforms. The prevailing design emphasis is directed toward expediting the immobilization process while concurrently enhancing the conductivity detection range within biosensing modules. Experimental trials and compositional testing aimed at optimizing morphology and design have yielded substantial improvements in the nano-sensing interface. Notably, these efforts have led to the integration of a more active surface area, thereby augmenting the sensitivity and efficacy of biomarker detection. The realm of nanocomposite development is particularly noteworthy, highlighting strides in enhancing charge carrier mobility and the emulation of low band gaps within material layers. One noteworthy example is the utilization of the carbon-based material known as ‘Graphdiyne.’ This material stands out due to its remarkable charge carrier mobility, facilitating efficient charge transport, and its judiciously selected band gap, which contributes to superior performance.^71^ In the context of detecting CA19-9 antigens, the conductivity detection limit achieved with Graphdiyne was remarkable, nearing 0.00012 U mL^−1^ within a concentration range spanning from 0.00051 to 199.99 U mL^−1^. Additionally, ionic liquids have garnered substantial attention in this arena, primarily owing to their compatibility with carbon nanotubes. Ionic liquids exhibit an outstanding electrochemical window, enhancing the sensitivity of the transduction mechanism, and thereby surpassing the capabilities of conventional detection methods.^72^ These materials also possess noteworthy optical properties, further expanding their utility in the analysis of biomarkers.

#### 2.2.2 Optical transduction

Recently, optical transduction-based biosensors have gained popularity in detecting cancer-related biomarkers with a high attainment ratio in terms of probability index. The optical transduction method uses light and wavelength-based spectra for various analyses. The optical transduction principle is based on reflection, refraction, grating, polarity, absorption, adoption, luminance ratio, interference, and compensation of light rays. Based on wavelength, the light signal has various categories, such as visible, infrared, and ultraviolet. In cell analytics, it has been observed that the spatial distribution of light helps to identify the cell morphology for detecting cancer-based target analytes. Optical transduction is a label-free mechanism that provides rapid and stable measurements.^73–75^

Uniyal *et al.* fabricated a frequency-based optical biosensor.^73^ The overall diameter of the biosensor was varied from 4 to 6 μm and 140 mm stretching length. The IgG layer is coated with polydopamine to trap anti-IgG. It has been observed that the concentration level of anti-human IgG linearly varied from 0 to 13.99 ng mL^−1^ with a sensitivity range of 49 mm. The recent advancement in photonic crystal fiber-based biosensors is also gaining popularity due to improved refractive index in relation to cancer-related antigens detection.^75^ The hybridization of photonic crystals with plasmonic metals such as gold, silver, and copper tremendously increase the biosensor's sensitivity. However, due to poor biomolecule adsorption, these sensors lack the authenticity of results.^76^

Optical transduction-based biosensors are highly regarded for their exceptional sensitivity, selectivity, reusability of assembly, and non-contact measurement capabilities. These attributes have led to their widespread adoption in the design of flexible wearable biosensors, where the need for versatile and reliable biosensing platforms is paramount. The optical transduction process comprises three fundamental components: a light source, an optical chamber, and a photodetector. Within this assembly, received optical signals are meticulously transformed into electrical signals. Importantly, this generation of electrical signals is entirely contactless, eliminating the need for traditional electrodes. The linear transfer of these signals is seamlessly integrated into a signal conditioning module. In the optical transduction module, electrodes are strategically employed to partition serum samples into sub-sections, facilitating the monitoring of various physio-optical characteristics of transmitted light rays.^77^ These optical signals dynamically interact with biomolecules within the electrode and transduction module assembly. The alterations in optical signal characteristics, such as changes in reflection, refractive index, fluorescence, and grating, are induced by chemical reactions between the target analyte and labeled substrate. The detection of cancer cell-based signals relies on diverse gene detection methods, encompassing genetic assembly formation and protein analysis. Noteworthy biomarkers, including Squamous Cell Carcinoma Antigen (SCCA), carcinoembryonic antigen (CEA), and prostate-specific antigen (PSA), have been successfully identified through these advanced biosensing techniques. It is important to highlight that the backbone of these biosensors lies in nanostructured materials.^78^ The integration of biocompatible nanomaterials in various structural configurations has yielded remarkable advancements in biosensing performance. A comprehensive overview of recent advances in optical biosensors is presented in Table 2.

**Table 2:** Response of biosensors based on optical transduction mechanism

Nature of material	Nanomaterial	Target analyte	Size (nm)	Range of detection	Limit of detection	Key features	Ref.
Organic	Gold electrode-MIP film	PSA	14–16	0.1–1 μm	0.1 μm	Because of the desirable biosensor qualities such as ease of use, cost, and sensitivity, novel technology has emerged as a method of employing optical features for cancer cell detection	79
	CuLnZnS-QDs	NSE	06–19	0.1–1.5 nM	0.03 nM	These biosensors are very sensitive, easy to build, inexpensive, and recyclable; yet, they are typically non-selective, and controlling the sensing process at high currents is difficult	80
	Hydrogel–Ag–Au nano frame	CA-125	40–60	1–50 μm	50 μm	Optical biosensors have superseded traditional techniques of detecting and quantifying CA-125 oncomarkers in biomedical research due to their capacity to perform multiplexed detection of several analytes and their real-time and direct monitoring capabilities	81
	Phthalocyanine-doped polystyrene shapes	CA-125	200–250	0.01–127 U mL^−1^	10^−4^ U mL^−1^	The sensing behavior of this metal organic framework (MOF) toward cancer biomarkers has been investigated using a variety of experimental and computational methodologies. Luminescence and electrochemical investigations of MOF sensors offered quick and cost-effective systems for detecting cancer biomarkers	82
Inorganic	Cadmium sulfide with AuNPs	NSE	05–20	20–103 fg mL^−1^	4 fg mL^−1^	The combination of CdS's light absorption and AuNP's plasmonic characteristics results in considerable signal amplification, allowing for the detection of extremely low concentrations of target analytes	83
	MNPs	PSA		15–21 CFU mL^−1^	14 CFU mL^−1^	This biosensor might be used as a novel clinical tool for early diagnosis and prognosis of prostate cancer. Furthermore, using the concepts utilized to design a biosensor, inexpensive and simple biosensors for early detection of a variety of human ailments, including heart disease, cancer, and infectious diseases, might be produced	84
	Au–Ag nanoclusters	PSA	10–25	0.4–36.5 U mL^−1^	0.29 U mL^−1^	The combination of AuNPs and AgNps improves the sensitivity and stability of the biosensor. The gold surface can easily change to attach antibodies or other PSA-specific recognition components, whilst the silver increases the sensitivity of the optical signal	85
	Gold nano frame with cadmium	CA19-9	05–12	1–70 μm	0.64 μm	This paper shows the successful creation of a highly sensitive and selective electrochemical biosensor for detecting CA19-9, a crucial biomarker for pancreatic cancer	86
Carbon allotropes	Europium-enriched GQDs	CEA	06–20	0.5–50 μm	0.31 μm	GQDs' strong electron transfer and facile biomolecule immobilization characteristics make them ideal for constructing effective (bio)analytical sensors. These photoluminescent QDs have exceptional biocompatibility, high current density, quick electron mobility, high water solubility, outstanding photochemical stability, and minimal cytotoxicity, making them dominant in the field of sensing	87
	AuNPs enriched graphene nano frame	PSA	09–16	0.51–0.4 μm	0.51 nM	This technology differs substantially from current peptide-based PSA detection methodologies. It has great promise for usage in laboratory investigations and clinical diagnostics because it involves only a basic sample handling process and a small investment in equipment	88
	Dendrimer wire with CQDs	SCCA	20–40	0.01–2 mM	0.01 μm	Depending on the material, intended fluorescence, and expected size, these carbon-based fluorescent nanoparticles can be manufactured with a number of chemical and natural chemicals and methods. Because of their unique adjustable optoelectronic capabilities, they display size-tunable emission, excitation-dependent emission, and solvent-dependent emission	89
	MWCNTs	NSE	05–15	0.05–1 ppb	0.05 ppb	This aptasensor demonstrated high repeatability, stability, and specificity, indicating a feasible technique for detecting NSE in clinical diagnostics	90

In recent developments within the realm of optical transduction-based biosensors, the exploration of photo-sensing has emerged as a focal point of research interest. This approach offers notable advantages, particularly in the facile detection of cancer biomarkers. A noteworthy study by Barve *et al.* has centered its attention on the identification of Neuron-Specific Enolase (NSE) as a pivotal biomarker derived from neuroendocrine cells.^89^ SE, an acid-based protease serum component, is extracted explicitly from neuroblastoma sources. Intriguingly, investigations have revealed the utility of the NSE biomarker in the early-stage detection of lung-related cancer cells. This innovative research underscores the potential of optical transduction-based biosensors in revolutionizing early cancer diagnosis through the precise identification of crucial biomarkers.

Wang *et al.* designed a net-based farmwork from the Au–Ag nanorods for target-doped photosensitive biosensors.^90^ In the experimental results, it was observed that the addition of a dopant in the transition composition could improve the sensitivity range and boost the amplification of the received signal with a high precision rate. Al Mahmud *et al.* designed a biosensor using the extract of cadmium-sulfate, which had an ultra-sensitive assembly for identifying PSA biomarkers from the sample.^91^ This biosensor assembly achieved ultra-sensitivity with a 1 pg mL^−1^ detection limitation. The response was approximately 100 times more sensitive than other categories having the same substances.

Zare *et al.* developed a QDs-based antibody-antigen assembly to detect CEA cancer biomarkers for effective quantitative analysis of cancer biomolecules on a spectrum form.^92^ Due to the proration in the quantum dots with nano frame structure, the sensitivity becomes very high with a detection limit below 80 fM. In immunoassay-based sensing methods, recent advances have been observed in label-free sensing to detect cancer-related biomarkers. Nanomaterial-based optical sensors can frame out the analyte chamber to produce a label-free spectrum of light to identify the target analyte.

Matar *et al.* used the fusion of SnS_2_-mpg-C_3_N_4_ to design a photochemical immunoassay biosensor to detect PSA biomarkers.^93^ This biosensor had an exceptional linear response concerning the target analyte, which varied from 55 fg mL^−1^ to 12 ng mL^−1^, and the sensitivity limit was near about 18 fg mL^−1^.

In addition, Foroozandeh *et al.* designed a nanoparticle (denoted as MoS_2_QDs-*g*-C_3_N_4_-CS-AuNPs) to build an optical sensor based on plasmon resonance with the limit of detection of 0.25 pg mL^−1^ and the linear range from 1 pg mL^−1^ to 24 ng mL^−1^.^94^ The spectroscopic methods are also embedded in the biosensors for the analysis of the biospectrum and the improvement of the quantification of target cells. Surface-enhanced Raman scattering is an ultramodern method based on the laser light spectrum.^95,96^ This assembly has four key modules (light source, monochromator, high-fidelity signal conditioning module, and photosensor) cascaded to study the target analyte. This method was suitable for capturing electrochemical reactions between antigen–antidot carriers with an exceptional detection limit. This scattering method exhibits excellent linear response toward studying metallic NP-based immunoassay biosensors. Surface-enhanced Raman scattering has recently gained popularity in biosensing devices due to its nanoscale fabrication. As per recent trends in optical transduction mechanisms, the amalgamation of both electrochemical transduction and optical transduction paves the way to improve biosensor designs in new horizons.^97^

#### 2.2.3 Photoelectrochemical transduction

With the advancement in the unification of nanomaterials, the strength of these biosensors is rapidly improvised in terms of linearity and sensitivity for quality detection of target analytes and its being in the way to obsolete the conventional immunoassay methods. The fusion of materials such as cadmium, silicon, gallium arsenide, and TiO_2_ with other materials helps to develop a dual transduction mechanism.^98^ Photoelectrochemical transduction is now becoming popular in analyzing complex cancer cell structures. The photoelectrochemical transduction method combines electrochemical and optical transduction mechanisms for critical sample analysis for multidimensional aspects. As per the latest trends, the spatial-temporal analysis can be possible in the biosensor module itself, and then with the help of an internet-of-things (IoT) based approach, the crisp results can be extracted without tampering with the biosensor from the target organ of the human body. The photoelectrochemical transduction-based biosensors are very fast in response with prodigious sensitivity and lower detection limit with significantly less interference between the sensing and detection units.^99^

The photoelectrochemical-based biosensors are working on the principle of irradiation of light signals. When the light assembly passes through the sample solution of the analyte, the strength of the photo signal varies. The electron and hole pairs are generated at the photo-sensing element as per the light received. It is worth using the low band gap semi-conducting materials to prepare photoelectrochemicals for improved results compared to the individual optical and electrochemical transduction-based sensors. This transduction mechanism opens a big pool of research in various dimensions, such as contactless detection, very low detection rate, and flexibility in making wireless. Recently, different exogenic materials such as Si, TiO_2_, CdS, and phosphate dots have been inspected and selected to make these kinds of sensors due to their spectacular performance in justifying hypothesis values on the probability index.^100–112^ Due to optical isolation between the electrode assembly and signal conditioning circuit, the output signal has no interference effect, and the use of light ensures the sensor's reuse on a periodic cycle. Table 3 shows the response of materials in relation to photoelectrochemical transduction.

**Table 3:** Response of biosensors based on photoelectrochemical transduction mechanism

Material	Nanomaterial base	Target analyte	Size (nm)	Range of detection	Limit of detection	Key features	Ref.
Organic	TiO_2_ crystals and PAMAM dendrimers	CYFRA 21-1	40–60	10–90 pg mL^−1^	3.3 fg mL^−1^	The nanoparticles were coupled with CYFRA21-1 to create a biosensor for monitoring nonsmall cell lung cancer due to their sensitive detection of CYFRA21-1	101
	2D porphrinic-COFs on AgNPs	PSA	50–100	0.5–100 ng mL^−1^	100 pg mL^−1^	COFs with large surface area and porosity, high electrical conductivity, and new interactions with bioactive substances have the potential to diagnose cancer (by biosensing and bioimaging)	102
	MIPs based on CNDs-embedded COFs	SCCA	250	0.025–0.4 mg mL^−1^	7 μg ML^−1^	The constructed biosensor demonstrated various benefits, including superb specificity, exceptional selectivity, and great stability. The suggested approach is suitable for the quick and sensitive detection of SCCA in human serum and shows potential for clinical usage	103
	Phthalocyanine-doped polystyrene spheres	CA-125	100–200	0.01–127 U mL^−1^	4 U mL^−1^	The significant suppression of the luminescence intensity of the Ni–phthalocyanine complex doped in PS matrix by varied concentrations of CA 125 was effectively used as an optical sensor for detecting CA 125 in ovarian disease blood samples	104
Inorganic	Pd–Ag–CeO_2_ core–shell NPs	NSE	40–110	0.4–99 ng mL^−1^	0.14 pg mL^−1^	The suggested nanocomposite structure demonstrated superior catalytic performance, increased specific surface area, and acceptable stability. The developed immunosensor demonstrated great sensitivity, adequate selectivity, and acceptable stability in detecting NSE	105
	Ag nanoplates	CEA	30–90	0.12–6 μg mL^−1^	42 pg mL^−1^	Given its simplicity, high sensitivity, outstanding repeatability, and specificity, the proposed biosensor can be deemed an effective diagnostic test for CEA measurement	106
	Nanoclusters of gold–silver	PSA	30–55	0.45–38 U mL^−1^	0.25 U mL^−1^	The findings suggest that integrating biosensors for cancer screening with serum PSA levels is a potential technique for enhancing prostate cancer diagnostic accuracy	107
	MNPs-tunneling magnetoresistance	NSE	25–75	14–1.3 × 10^9^ CFU mL^−1^	13 CFU mL^−1^	The suggested idea of a simple tunnel magnetoresistance (TMR) sensor for real-time detection of MNPs has considerable potential applications in cancer cell localization by magnetic particle detection in tissue and cancer treatment *via* magnetic hyperthermia	108
Carbon allotropes	ZnFe_2_O_4_–Ag/rGO nanocomposites	CEA	40–80	1–200 ng mL^−1^	0.98 pg mL^−1^	The nanohybrid's exceptional high catalytic capacity may allow it to replace current target molecule identification methods	109
	Graphene	PSA	30–90	0.06–101 ng mL^−1^	0.04 ng mL^−1^	The suggested immunosensor demonstrated remarkable performance, with high sensitivity, selectivity, and stability. On the one hand, this form of electrochemiluminescent immunosensor should be practical for the field of cancer biomarker detection and other life science domains	110
	Europium-enriched GQDs	CEA	45–110	0.6–49 μM	0.34 μM	A novel photoluminescence (PL)-based assay for the detection of CEA has been presented, which is based on the graphene-quantum-dot (GQD) surface for Eu^3+^ ions. The graphene-like structures, paired with QD-like optical characteristics, point to GQDs' potential as flexible instruments in analytical science and biology	111
	Magnetic ND/GO	PSA	50–90	5–249 ng mL^−1^	1.50 ng mL^−1^	The developed biosensor could detect extremely low levels of PSA	112

Recently, Fan *et al.* designed a photoelectrochemical biosensor using the TiO_2_/PpIX/Ag–Cu_2_O composition. This assembly was beneficial in identifying the CYFRA 21-1 biomarker associated with lung cancer. The hydrothermal method was used to fabricate TiO_2_ nanomaterials on the protoporphyrin IX (PpIX) molecules and Ag–Cu_2_O. This assembly has an excellent response in the visible light spectrum, so the photoelectric chemical properties are improved from other families of the same biosensors group. As per the results, this assembly attains the regenerative mechanism for electron–hole pairs, which increases the photocurrent amount from the previous threshold level. This assembly provides a high-sensitivity response with a wide range of linearity in the photospectrum.^112^

Esfandiari *et al.* developed a biosensor assembly to diagnose BC-related MDA-MB-231 biomarkers. The photoactive material composition of heterostructures (CdS/Bi_2_S_3_HSs) was fabricated to prepare the modular biosensor in cascading with signal amplification strategy for immune identification between epidermal receptors. The results show that this assembly has an excellent response during photoelectrochemical reactions. This biosensor exhibits improved sensitivity and adsorption response for recognizing the MDA-MB-231 biomarker. The linear response of this sensor module varies from 98 to 3 000 500 cells per mL with six cells per mL detection range. The signal-to-noise ratio of this biosensor is 3.^113^

Yu *et al.* have prepared an improved and sophisticated label-free photoelectrochemical immunoassay biosensor using the photoactive nanomaterial composition of Bi_2_WO_6_/BiOBr to detect prostate-specific antigens. This heterojunction-based biosensor composition attains various properties, such as improved photocurrent response due to immobilized dopamine current. In the optimal testing conditions, the sensor produces an improved linear response ranging from 1 pg mL^−1^ to 51 ng mL^−1^.^114^

The instrumentation-based parameters such as stability, selectivity, reproducibility, and the ratio of signal to noise of this assembly pave the way to develop real-time responsible sensors. The study of Han *et al.* led to the development of ultra-thin and diffusion-based immune sensors to detect prostate-specific antigens in the human body. The authors used Fe-loaded Bi_2_O_2_S nanosheets to diffuse photoanode crystals with a photoelectric transduction mechanism. Response of the split incubation reaction in the signal probe (PB-mAb_2_) helps to mark the presence of target PSA antigens. The cost of this assembly is far less than other material-based assemblies. They achieved a wide range of linear responses ranging from 0.1–105 ng mL^−1^ with the detection limit near 35 pg L^−1^. This sensor assembly leads to the designing and developing of very low-cost and real-time sensors to identify cancer-based cells.^115^

#### 2.2.4 Piezoelectric transduction

This transduction mechanism is developed by using piezoelectric materials in MEMS-based biosensors. The electromechanical properties of these materials exhibit various properties, such as label-free sensing of cancer cells with ultra-low detection limits.^116,117^ The current research community is moving towards developing a bio-flexible and wearable sensing mechanism, which is only possible with simple and effective electromechanical assembles. The piezoelectric transduction is well-known for its simple yet aggressive fabrication properties to detect cancer-related biomarkers. The current research community is moving towards developing a bio-flexible and wearable sensing mechanism, which is only possible with simple and effective electromechanical assembles.^118^ The microcantilever mechanism is very popular for detecting the target analyte with a lower detection limit. The piezoelectric transduction-based biosensors are working on the principle of quartz crystal microbalance resonance frequency balancing. Nonpolarized materials (anisotropic by nature) such as aluminum phosphate, aluminium nitride, and zinc oxide are used to fabricate piezoelectric biosensors. When this fabricated assembly is charged with the specific alternating current (AC) signal voltage of frequency of 1 to 10 Hz, then a mass-based restraining is originated on the plate areas of a piezoelectric crystal. When it meets blood plasma, the oscillation behavior suddenly changes regarding the target analyte. This transduction mechanism has many applications in biotechnology-based tissue engineering and genetic studies due to its rapid response with satisfactory instrumentation characteristics such as label-free sensitivity, Signal-to-noise ratio, repeatedly, reusability, and low detection limit.^119^ The piezoelectric materials fusion with other nanomaterials classes is shown in Table 4.

**Table 4:** Response of biosensors based on piezoelectric transduction mechanism

Material	Nanomaterial base	Analyte	Size (nm)	Range of detection	Limit of detection	Ref.
Organic	Gold surface modified nano film of 2-hydroxyethyl methacrylate ethacryloylamido aspartic acid	PSA	24 nm	0.1–1.25 nM	0.03 nM	120
	Bilirubin-imprinted nanofilm with 2-hydroxyethyl methacrylate methacryloylamido aspartic acid	CEA	40–90	1–50 μg mL^−1^	0.45 μg mL^−1^	121
	Zr-amide porphyrin-based COFs	CA-125	40–75	5–60 pM	2.3 pM	122
	CuInZnS-QDs with polydopamine and AuNPs	PSA	30–50	0.1–1.5 nM	0.03 nM	123
Inorganic	AuNPs	CA-125	40–110	10–99 pM	2.4 pM	124
	MNPs	PSA	60–112	14–1.3 × 10^9^ CFU mL^−1^	13 CFU mL^−1^	125
	Nanoclusters of gold–silver	CEA	30–90	0.6–38 U mL^−1^	0.4 U mL^−1^	126
	Cadmium sulfide AuNPs	CA-125	30–65	20–103 fg mL^−1^	4 fg mL^−1^	127
Carbon allotropes	CNTs-amended graphite electrode	CEA	25–75	0.05–1 nM	18 μM	128
	CQDs	PSA	40–78	0.714–4.286 ng mL^−1^	Less than 1 ng mL^−1^	129
	Carbon–silver-based multiwalled nanotubes	CEA	80–140	0.25–250 ng mL^−1^	0.08 ng mL^−1^	130
	Gold–silver–graphene hybrid nanosheets	PSA	40–110	0.002–201 ng mL^−1^	0.45 pg mL^−1^	131

Bakhshpour *et al.* developed a multi-walled carbon nanotube (MWCNT)-based biosensor. In this transduction mechanism, two electrodes were fabricated on an aluminum nanoplate, and then the alternating voltage was applied to observe the vibration-based signal at the actuation point.^130^ The measured physical indication was very low, but when the frequency of the applied signal was changed, at a point, the strength of the physical signal increased to represent the target analyte during immunoassay trials. When the result of this biosensor was calibrated with a conventional ELISA test, the 95% confidence interval was reported on the probability index. Hence, this simple mechanism indicates the importance of piezoelectric transduction in relation to the development of biosensors in cancer diagnosis to the research community. The various parameters of this biosensing assembly, such as low fabrication cost, real-time response, high sensitivity, ultra-lower detection limit, reproducibility, and flexibility in the electrode assembly, attract the researchers to find new horizons.

Lino *et al.* developed fan-shaped and circular-shaped biosensors by using flexural plate-wave-based materials such as silico, ferromagnetic metal, and quartz-based multilayer floating structures to detect CEA.^131^ The structure was developed to implant a silicon-grooved reflective grating property for balancing the resonance effect. In the trials, they achieved a minimum detection limit of 6 ng mL^−1^, a time response of 8 min, and a high linear sensitivity response of 50 to 60 cm g^−1^. With the advancement in photolithography-based fabrication of nanomaterials, multi-layered biosensor electrodes with piezoelectric transduction mechanisms were developed.

Villarim *et al.* developed a fusion of plasmon resonance-based biosensors in association with the piezoelectric effects of metals to reduce the reaction time inside the assembly for fast response.^132^ The enhanced nanolayers of AuNPs and piezoelectric material NaNbO_3_ were fabricated to detect the immune incubation process of target PSA from the plasma. Using the density function spectrum, the confidence interval of measurement regarding linear response and sensitivity was 97% concerning the ELISA test for target PSA. This assembly sets a benchmark for the quick and meticulous detection of cancer-related proteins. The electrochemical methods adopted to study cancer biomarkers have limited transfer of electrons from the sensor to the actuation module. There is a need to resize the platforms for effect measurements.

Li *et al.* developed a piezo-phototropic-based flexible biosensor for recognizing PSA from the cancer-associated biological fluid.^133^ In addition to this transduction mechanism, a multilevel signal enhancement scheme was adopted to promote the detection of ascorbic acid from the substrate, enhancing the sensitivity limit of this biosensing module. Unlike the electrochemical and optical transduction mechanism, this assembly promotes the transfer of physical information about chemical reactions on an ultrasonic signal level, which is why the label-free detection was done with a very low interface level between the cascaded modules. This biosensor assembly achieved the detection sensitivity of PSA from 0.02–40 ng mL^−1^.

#### 2.2.5 Aptamer transduction

With the advancement in immunoassay methods, biomarker-based antibodies are developed to trap the target antigen from the serum of a cancer-based sample solution.^134,135^ By this approach, the label-based biomarkers can be detected without efforts to transduce the signal. Aptamer biosensors use oligonucleotide aptamers with folding-based binding to detect the specific ions, proteins, and molecules-based biomarkers. They have a tremendous trap to identify the biomarker with a sequence of nucleic acids. During the electrochemical process, these aptamers trap the availability of specific biomarkers and then reflect the information in the form of change in amperometry, impedimetric, voltammetric, and photoelectrochemical mechanisms.

To detect the circulating tumor cells (CTCs), Chi *et al.* developed a Bi_2_O_2_S nanoflowers, which was fabricated along with 45 nm sized AuNPs.^136^ The composition of AuNPs/Bi_2_O_2_S coated on ITO electrode with mercapto-group functionalized aptamer (SH-Apt). During the photoelectrochemical process, it has been observed that the MCF-7 cells were specifically trapped on the prepared hydrothermal-based composition, likely on the surface of SH-Apt/AuNPs/Bi_2_O_2_S/ITO. This biosensor produces a linear response ranging from 50 to 6 × 10^5^ cells per mL with a detection limit of 17 cells per mL. This study helps to test the serum on a clinical level. This configuration indicates the importance of the aptamer transduction mechanism in the detection of specific tumor cells related biomarkers. The response of biosensors based on the aptamer transduction mechanism is shown in Table 5.

**Table 5:** Response of biosensors based on aptamer transduction mechanism

Material	Nanomaterial base	Target analyte	Size (nm)	Range of detection	Limit of detection	Ref.
Organic	2D porphrinic-COFs on AgNPs	CEA	80–100	0.5–100 ng mL^−1^	100 pg mL^−1^	137
	TiO_2_ crystals and PAMAM dendrimers	PSA	40–89	10 ag mL^−1^ to 90 pg mL^−1^	3.3 fg mL^−1^	138
	4-Aminobenzoic acid/MWCNTs	CA-125	25–30	0.25–10 μM	0.2 μM	139
	Gold electrode-MIPs film	NSE	14–16	0.1–1 μM	0.1 μM	140
Inorganic	Gold–graphene NCg	PSA	20–100	6–1.6 × 10^3^ μM	0.5 μM	141
	Nanoclusters of gold–silver	PSA	80–120	0.5–36 U mL^−1^	0.4 U mL^−1^	142
	AuNPs	CEA	45–95	10–100 pM	2.4 pM	143
	SH-Apt/AuNPs/Bi_2_O_2_S/ITO	CTC	40–95	50 to 6 × 10^5^ cell per mL	17 cells per mL	144
Carbon allotropes	AuNP-rGO	PSA	50–150	0.03–520 ng mL^−1^	0.041 ng mL^−1^	145
	ZnFe_2_O_4_–Ag/rGO nano-frame	CEA	50–110	1 pg mL^−1^–220 ng mL^−1^	0.88 pg mL^−1^	146
	Graphene oxide-chitosan-ferrocene nanoplates	SCCA	20–100	0.001–9 μg mL^−1^	0.35 ng mL^−1^	147
	Au–Pt-vertical graphene electrode	NSE	40–65	1 fg mL^−1^–104 ng mL^−1^	0.8 fg mL^−1^	148

### 2.3 Comparative analysis of different transduction methods

The inherent miniaturization, mass production, and low cost of electrochemical transduction make it stand out. However, before portable, reliable, and user-friendly point-of-care biosensors for cancer diagnosis through the detection of circulating miRNA expression profiles become a reality, there are still several significant obstacles to overcome. Additionally, as a result of these difficulties, there is currently no commercial electrochemical biosensor for circulating miRNA analysis to the best of our knowledge; as a result, more work has to be done soon on validation, clinical assays, and commercialization.^149^ Currently, electrochemical detection is dealing with many issues that need to be resolved to create a precise and sensitive cancer identification technique. Li has examined the sensitivity ranges of many methods for determining the least residual illness of hematologic neoplasms.^150^

Hematological neoplasms can be identified with a better degree of sensitivity thanks to electrochemical biosensors. For electrodes intended for single use or for repeated usage, the stability of the biosensor is a crucial component. An alternative but more important topic is how to appropriately assess actual samples *in vivo* for the advancement of electrochemical biosensors in the future. These challenges have thus far kept these approaches mostly in the experimental realm, preventing their widespread clinical use. The ideal *in vivo* sensor should be stable, sensitive, safe, and compatible with cells and tissues. An electrochemical biosensor needs to possess exceptional selectivity and be capable of detecting several tiny molecules, including aptamers and folic acid, as individual components. Furthermore, a powerful method in bioresearch to achieve an incredibly sensitive and discriminating identification of various compounds is the combined use of the unique identification powers of aptamers and the catalytic ability of enzymes. The identification of electroactive substances is substantially facilitated by enzymes associated with biomolecules.^151^

Non-toxic, low-reactivity nanomaterials that do not elicit an immune response can be used to optimize the biocompatibility of these biosensors. Suppose it is shown that nanoparticle-based electrochemical biosensors are very sensitive and effective detection instruments in *in vivo* experiments as well. In that case, they may eventually emerge as the standard way for diagnosing leukemia.^152^

Although aptasensors provide many benefits, there are still issues with aptamers that restrict the practical uses of these sensors. The applicability of aptasensors in complex biological systems is limited because the immobilization of aptamers onto surfaces might alter the aptamer conformation, which is further influenced by the makeup of the binding environment. There are still difficulties; for example, poorly designed proteins may interact non-specifically with aptamers and cover the analyte's unique binding site. Moreover, aptamers and the nucleic acids found in biological fluids may hybridize to modify the aptamer's conformation and, in turn, the analyte's binding site.

While some of the issues listed above are being addressed by developing research in the field, other areas of interest include novel techniques like the creation of aptamer-based micro-/nanochips for therapeutic applications. Additionally, aptamer microfluidic/wearable devices are anticipated to be crucial in the early diagnosis of cancer. Aptasensors have reportedly been used for cancer diagnosis in a number of different ways. These biosensors' aptamers are engineered to offer exceptional selectivity and enhanced sensitivity for the early detection of key cancer kinds, including those that impact the prostate, breast, and lung tissues of humans. Diagnostics is greatly aided by some biomarkers that are often expressed on the surfaces of these cancer cells. While many of the aptasensors that have been published are just electroanalytical and have no therapeutic use, the development of methods based on ratiometric electrochemical sensors can enhance the sensitivity, specificity, and output of cancer biomarkers. Furthermore, to build point-of-care testing (POCT), novel methods with enhanced sensitivity that are scalable and specific enough for early-stage cancer diagnosis are required. The next stage that will spur the commercial deployment of aptasensors for broad cancer diagnoses is the development of POCT. Aptasensors have several benefits, such as a low cost and relatively quick analysis time, which makes them extremely promising for clinical applications. However, there are also some drawbacks, such as the lack of long-term stability of bioelectronic chips. Thus, with more study, aptamer-based biosensors can be created as strong instruments for the accurate and efficient identification of biomarkers of different cancer cells in physiological fluids.^153^

Optical biosensors, including metamaterials, evanescent waves, colorimetric detection, and surface plasmon resonance, have been used in the detection of cancer. It has been demonstrated, meanwhile, that conventional techniques, such as optical coherence tomography, surface-enhanced Raman spectroscopy, colorimetric detection, and reflectometric interference petrography, call for a skilled operator to oversee the data that is being received. Furthermore, colorimetric detection is primarily dependent on the size, shape, and material type of the nanoparticles utilized, which may lead to a high degree of fault probability, even if it is simple to set up and the necessary equipment is readily available. The SERS method is likewise linked to the same drawbacks. Despite being quick, safe, and non-invasive, OCT is expensive and mainly utilized for diagnosing retinal conditions. Furthermore, temperature changes do not affect the RIfS approach. Conversely, novel methodologies such as SP PCF, slot waveguides, and metamaterials rely on quantifying the shift in resonance wavelength/frequency in accordance with the blood specimen being examined. As a result, these techniques can identify cancer early, are label-free, and do not require the assistance of an expert to do the analysis. It is also important to remember that slot biosensors can provide strong field confinement in the analyte under study, which can lead to great sensitivity. Additionally, the SP PCF sensors offer great sensitivity and design freedom. Moreover, depending on the analyte being studied, metamaterial biosensors can generate sharp resonance, which can be detected with great precision.^154^

Another exciting prospect for the future of optical nanobiosensors is *in vivo* fluorescence imaging of circulating cancer biomarkers. However, further research on the cytotoxicity of the nanoparticles will be necessary. For the time being, the emphasis will be on facilitating the synthesis and functionalization of those particles as well as enhancing their fluorescence through the combination of other enhancing processes or an increase in quantum yield.^155^

Many modifications are known for piezoelectric biosensors, which have been experimentally shown to be dependable. The primary disadvantages of piezoelectric biosensors are their reliance on sample preparation and intricate pretreatment procedures; however, the experimental results are encouraging, and a shift in this situation is anticipated in the near future. The development of low-cost piezoelectric materials and the ability to produce targeted materials in large quantities, such as oligonucleotides, nanoparticles, and imprinted polymers, provide promising indications for future advancements in this sector.^156,157^

## 3. Biosensors in different cancer diagnostic measurements

**Fig. 3:**
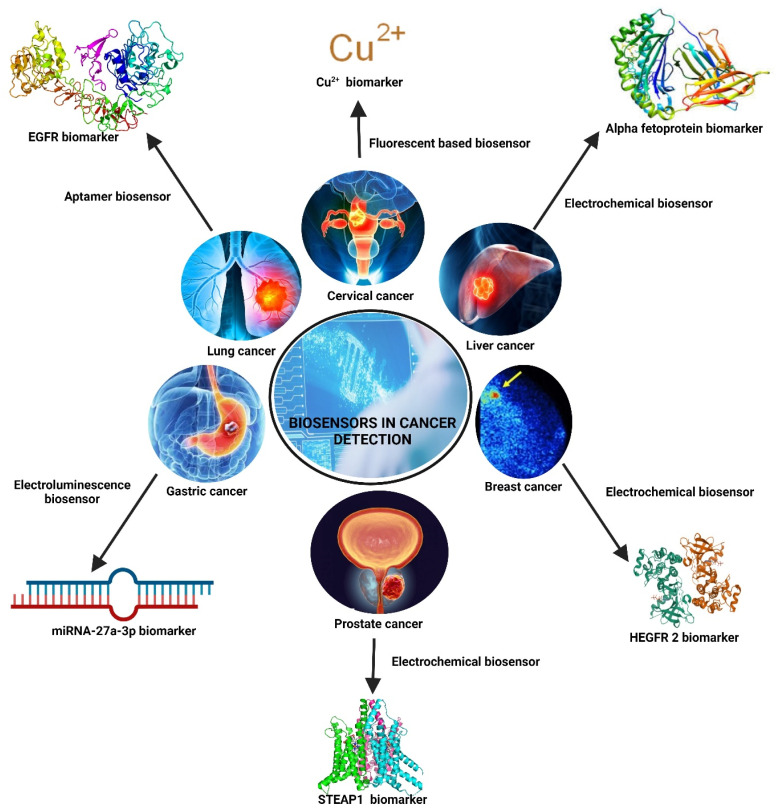
Biosensors in the detection of various cancer biomarkers.

As per the WHO report of 2020, more than 15 million people worldwide lose their lives yearly due to various cancer-causing agents such as pathogens and carcinogens. However, each category of cancer is a matter of concern, but as per the reported number of cases, the mortality rate is higher in specific types of cancer diseases. Among the cases, the most significant number of cases are reported concerning various body organs, such as lungs, liver, gastric, breast, prostate, and cervical cancer (Table 6). The state of the art in biosensing plays a pivotal role in diagnosing these types of cancers (Fig. 3).

**Table 6:** Schematics on diagnostic measurements of different cancers by biosensors

Targeted cancer	Studies	Key findings	Ref.
Lung cancer	Evaluating the efficacy and cardiotoxicity of EGFR-TKI AC0010 with a novel multifunctional biosensor	AC0010 (10 μM) effectively inhibited EGFR-T790M mutant NSCLC cell lines without compromising cardiomyocyte viability	158
		Within 1 h, 10 μM AC0010 increased frequency and decreased amplitude of extracellular field potential along with mechanical beat	
	A highly sensitive sensor for carcinoembryonic antigen based on AlGaN/GaN high-electron-mobility transistors	CEA antibodies were immobilized in the sensing area of AlGaN/GaN high-electron-mobility transistors, allowing for specific identification	159
	Vangl-dependent Wnt/planar cell polarity signaling mediates collective breast carcinoma motility and distant metastasis	Vangl-dependent Wnt/PCP signaling enhances breast cancer collective cell migration regardless of tumor subtype, indicating a critical function for this system in breast cancer development	160
		This sensor can interpret Wnt/planar cell polarity (Wnt/PCP) metastatic signals to assess the etiology and symptoms of lung cancer	
Liver cancer	Electrochemical immunosensor based on Fe_3_O_4_/MWCNTs-COOH/AuNPs nanocomposites for trace liver cancer marker alpha-fetoprotein detection	The combination intended to improve the sensor's electrochemical performance	161
		The immunosensor performed well in identifying AFP in clinical samples, showing its potential for real-world use in early liver cancer detection	
	Well-ordered Au nanoarray for sensitive and reproducible detection of hepatocellular carcinoma-associated miRNA *via* CHA-Assisted SERS/fluorescence dual-mode sensing	This sensing technique can detect the target miR-224 over a large linear range (1 fM to 1 nM)	162
		This biosensor, with its high specificity and anti-interference capacity, can distinguish target miR-224 from other interference miRNAs	
		This biosensor can differentiate HCC cancer subjects from normal ones, monitor HCC patients before and after hepatectomy, and guide the various Barcelona clinic liver cancer (BCLC) phases	
	Self-powered logically operated fluorescent detection of hepatitis B virus (HBV)	The suggested biosensor detects HBV-DNA at a threshold of 1 fM in human serum	163
Gastric cancer	A novel bimetallic MXene derivative QD-based ECL sensor for miRNA-27a-3p detection	The biosensor demonstrated a strong linear connection between ECL intensity and miRNA-27a-3p concentration over a wide range of 1 fM to 10 nM, with a detection limit of 1 fM	164
		The sensing system, which included Mo_2_TiC_2_ QDs, SnS_2_ nanosheets, and lipid bilayers, exhibited a high potential for therapeutic applications	
	Novel sensitive electrochemical immunosensor development for the selective detection of HopQ *H. pylori* bacteria biomarker	The use of MWCNT/AuNP nanocomposite enables for antibody covalent bonding, which improves SPCE performance	165
		It is promising to develop cost-effective biosensors compatible with point-of-care test technologies for *H. pylori* detection in order to reduce its impact on mankind	
		This study is the first to disclose the application of HopQ as a biomarker for *H. pylori* in an electrochemical immunosensor, as well as the dissociation constant of HopQ/HopQ-Ab interaction	
	Photofuel cell-based self-powered biosensor for HER-2 detection by integration of plasmonic-metal/conjugated molecule hybrids and electrochemical sandwich structure	The localized surface plasmon resonance impact of AuNPs may clearly improve the separation efficiency of photo-generated electron/hole pair, which was favorable to the sensitivity and stability of PFC-SPB	166
		Using the open circuit voltage (EOCV) as the output signal, the PFC-SPB can detect HER-2 in the 0.1–500 pg mL^−1^ range, with a low detection limit of 0.02 pg mL^−1^	
Breast cancer	Development of an electrochemical biosensor for the detection of mammary gland carcinoma using molybdenum enhanced poly taurine nano-biofilms confirmed pathological findings	According to the desired collaboration with pathological studies, the developed biosensor is an appropriate diagnostic tool for the examination of HER-2 positive actual samples in clinical laboratories	167
	A colorimetric biosensor to track Trop-2 status of tumor cells for diagnosis of breast cancer	A dual-aptamer-assisted biomimetic capture approach achieves high capture efficiency while preserving the viability and original phenotypic of captured cells, allowing for precise Trop-2 status analysis	168
		The high intrinsic peroxidase activity and excellent targeting ability of Trop-2-specific aptamer-linked TDN-PCN-222 (Fe) allow for the specific detection of Trop-2-positive tumor cells with a limit of detection (LOD) of 10 cells per mL, as well as the visual tracking of tumor cell Trop-2 status	
	Faraday cage-type ECL biosensor for the detection of circulating tumor cell MCF-7	Under ideal experimental circumstances, the limit of detection was 3 cells per mL	169
		The sensor was capable of detecting genuine human blood samples, marking the first report on the identification of entire circulating tumor cells using a Faraday cage-type electrochemiluminescence biosensor	
Prostate cancer	Development of a novel electrochemical biosensor based on plastic antibodies for detection of STEAP1 biomarker in cancer	The biosensor demonstrates a linear detection range from 130 pg mL^−1^ to 13 μg mL^−1^, indicating its potential for sensitive and reproducible detection of STEAP1 in medical and clinical research applications	170
		The biosensor employs MIPs, synthetic receptors designed to imitate natural antibodies, resulting in great specificity for the target biomarker	
	A sensitive label-free biosensor based on Ag_2_S-sensitived Bi_2_WO_6_/BiOBr heterojunction for photoelectrochemical immunoassay of prostate specific antigen	Antigen and antibody are precisely mixed to provide quantitative PSA detection based on current changes at varying antigen concentrations	171
		Under perfect experimental circumstances, the PEC immunosensor exhibits an excellent linear relationship between 1 pg mL^−1^ and 50 ng mL^−1^, with a detection limit of 0.084 pg mL^−1^	
	Suspended CNTs/MoS_2_ heterostructure field effect transistor for high performance biosensor and serum biomarker detection	The suspended CNTs/MoS_2_ FET demonstrated sensitive and reliable detection capabilities, with significant promise for quick clinical assessment and biomarker monitoring	172
Cervical cancer	A new Nbd-based probe for specific colorimetric and turn-on fluorescence sensing of Cu^2+^ and bio-imaging applications	A fluorescence imaging investigation demonstrates that probe AH can identify Cu^2+^ in live HeLa cells, locust Malpighian tubules, and zebrafish	173
	An ultrasensitive electrochemical DNA biosensor for monitoring human papillomavirus-16 (HPV-16) using graphene oxide/Ag/Au nano-biohybrids	The biosensor detected a qualitative difference between the probe and the target HPV-16 DNA	174
		The designed biosensor detected HPV-16 with a high sensitivity of 0.54 mA aM^−1^	
	Design and development of an electroanalytical genosensor based on Cu-PTCA/rGO nanocomposites for the detection of cervical cancer	The genosensor demonstrated good selectivity for HPV-16 target DNA, with no appreciable interference from non-target DNA sequences	175

### 3.1 Biosensors in lung cancer detection

Jiang *et al.* experimented with evaluating the response of AC0010-based aptamer biosensors for detecting lung cancer-related biomarkers such as Non-Small Cell Lung Cancer (NSCLC), one of the main cancer-causing factors responsible for mortality worldwide. This biosensor architecture integrates interdigital and microgrid electrodes to quantify the label-free response of AC0010-induced NSCLC inhibition. This biosensing assembly can produce the output in terms of real-time response on a monitoring screen by a non-invasive way of implanting a biosensor in the human body. The embedment of the digital electrode in the biosensor assembly is responsible for a linear response of 0.8–119 ng mL^−1^ with a detection range of 106 pg mL^−1^.^158^

Huang *et al.* developed an effective biosensor for carcinoembryonic antigen detection, known as a serum-based biomarker in lung cancer diagnosis. This sensor is designed in a nanoscale transistor form using AlGaN/GaN material due to various miraculous properties such as high levels of electron mobility and real-time response in the transduction assembly. This optical sensor can produce a linear response from 0.03–125 U mL^−1^ with a limit range of 3 to 3.4 U mL^−1^. Due to the use of AlGaN/GaN material, the cost of this biosensor becomes much lower than the other sensing mechanisms in this category, with an improved response on a time scale.^159^

VanderVorst *et al.* developed a multi-functional biosensor assembly to scale out the progress in breast tumor cells. This sensor can translate the progress in metastasis signals such as Wnt/planar cell polarity (Wnt/PCP) to measure the advanced status of lung cancer-related causes and symptoms. This biosensor mechanism focuses on associating tetraspanin-related proteins, such as Vangl1 and Vangl2, on measuring breast tumor cell activities. The results show that the Vangl1 and Vangl2 proteins can detect the cytoskeletal regulator RhoA-based leader cells in terms of tumor growth.^160^

### 3.2 Biosensors in liver cancer detection

Wu *et al.* developed an advanced electrochemical biosensor for detecting liver cancer biomarkers at very early stages of progression. In this sensor assembly, authors used iron tetroxide-based nanotubes with the fusion of carboxylate carbon and AuNPs to scale out the alpha-fetoprotein level in the human subject's liver. The working strategy of this sensor assembly is very sophisticated in generating the electrical signal spectrum to replicate the monoclonal antibodies of alpha-fetoprotein level. This sensor's response was linear and had a detection limit of 1.06 pg mL^−1^.^161^

Huang *et al.* developed an optical biosensing assembly to detect serum-based hepatocellular carcinoma and related miRNA, specifically miR-224. This sensor assembly contains well-arranged Au nanoarrays with substrate-coupled hairpin assembly for target-catalyzed serum. The miRNA was detected with a linear range of 1.0 fM to 1.3 nM based on the fluorescence-based dual-mode detection process. The detection limit of this sensor varied from 0.31 to 0.34 fM. This sensor assembly has ultra-sensing capability due to the integration of a catalyzed hairpin assembly amplification unit.^162^

Imbriano *et al.* developed a fluorescent-based smart digital biosensor for identifying hepatitis B virus DNA-based enzymatic logic network for cancer applications. This optical sensor contains an AND-based logic gate having two input electrodes (input 1: alanine aminotransferase (ALT) and input 2: lactate dehydrogenase (LDH)) and one output in the form of lactate (quenching DNA). In this assembly, as per the amount of measurement, the specific electrode generated the electric signal to present the quantity of quenching DNA labeled with BHQ2 or FAM. The detection limit of this sensor varied from 0.27 to 0.33 fM with a high level of sensitivity. The working mechanism of this sensor is a benchmark for upgradation in digital sensor development.^163^

### 3.3 Biosensors in gastric cancer detection

Li *et al.* developed an electrochemiluminescence biosensor to detect the miRNA-27a-3p biomarker for stomach-related cancer consequences. The authors developed the biosensor assembly using the QDs of bimetallic MXene derivative material and SnS_2_ nanosheets for better luminescence output with stability in electric field potential related to the oxidation process. This sensing mechanism produced excellent reproducibility of an analyte with linear response. The limit of detection was even less than 1 fM. So, overall, for detecting miRNA-27a-3p biomarkers, the fusion of Mo_2_TiC_2_ QDs and nanosheets of SnS_2_ composition plays a pivotal role.^164^

Jaradat *et al.* developed a photo-voltammetry-based immunoassay biosensor for detecting *Helicobacter pylori*-based HopQ protein biomarker for early detection of cancer symptoms in gastrointestinal saliva samples of human subjects. The biosensing assembly was developed on a thin carbon sheet coated by MWCNT-COOH and AuNPs. The sensor mechanism was based on electrochemical oxidation-based impedance spectroscopy to present the target analyte. The sensor had excellent linear response ranging from 10 pg mL^−1^ to 100 ng mL^−1^ range with a detection limit of 2 pg mL^−1^.^165^ This sensor had shown good response in selectivity, stability, and reproducibility for detecting *H. pylori*-based HopQ protein, a potential biomarker for insight into gastro-related cancer diseases.

Bai *et al.* a photo fuel-based self-powered electronic biosensor for breast and gastric cancer detection. In this sensor assembly, they sandwiched the QDs of plasmonic gold and hybrid photoanode-based organic semiconductor layers to detect human epidermal growth factor receptor-2 (HEGFR-2), a potential key biomarker for detecting early breast and gastric cancer symptoms. As per the resonance effect of localized surface plasmon-based electron–hole pair generation, the photo spectrum was developed to scale out the target analyte. This assembly provides an excellent linear response ranging from 0.2–510 pg mL^−1^ with a detection limit of 0.03 pg mL^−1^.^166^ The self-charging of biosensors is one of the primary vital concepts that set the benchmark for development in a system on a chip module.

### 3.4 Biosensors in breast cancer detection

Nasrollahpour *et al.* developed an electrochemical synthesis-based biosensor framework for the early detection of BC-related symptoms in female human subjects. The authors fabricated the biosensing assembly using molybdenum trioxide/polytaurine nanofilms to process the serum from the breast to detect the target analyte, HEGFR-2. This sensor could assess sample fluid regarding morphological and elemental progression in cancer-causing agents. The sensor's response time was significantly less and had a high reproducibility range. The linearity range of this biosensor varied from 0.1 ng mL^−1^ to 0.000001 ng mL^−1^, with a significant detection limit of 0.000001 ng mL^−1^.^167^ This sensor has proved reliable in using biomaterials for clinical applications.

Zeng *et al.* developed a colorimetric biosensor processing of track Trop-2 biomarker for early detection of symptoms of BC. This aptamer-based biosensor has excellent specificity and sensitivity for the target analyte with label marking of the target analyte. This sensor was fabricated using organic material-based nanoplates of Fe-based metal with a tetrahedral DNA-based nanostructure to process the target analyte. This biosensor produced excellent linearity with a 10 cells per mL detection limit adopted for BC-based clinical trials.^168^

Zhang *et al.* developed an electrochemiluminescence-based biosensor with Faraday cage technology to detect the MCF-7 BC cells in an early stage of development. In this sensor mechanism, the Faraday cage consists of two consecutive sections. The capture section and signal unit were designed by Fe_3_O_4_ NPs and GO-PTCA nanosheets, respectively, for photochemical synthesis. This aptamer-based material gave a very sensitive response to detecting the target analyte in MCF-7. The detection limit of this sensor was observed to be 3 cells per mL.^169^

### 3.5 Biosensors in prostate cancer detection

Carvalho *et al.* developed an electrochemical biosensor using CNTs and dendritic platinum NPs to detect STEAP1 biomarkers for prostate cancer treatment. In this biosensor, the oxidative modification of screen-printed electrodes in conjugation with lysates of neoplastic detects the prostate antigens. This electro-polymerization-based biosensor has an excellent linear response of 131 pg mL^−1^ to 14 μg mL^−1^ with a detection range of 0.3 pg mL^−1^. This sensor has produced supportive instrumentation factors, *i.e.*, reproducibility, selectivity, and precision for clinical applications and system-on-chip progression.^170^

Chang *et al.* developed a photoelectrochemical-based biosensor for the identification of prostate-specific antigens. This sensor was fabricated using Bi_2_WO_6_/BiOBr nanomaterials in conjunction with an Ag_2_S agent for sensitization. The current response of this sensor was based on the change in concentration of PSA antigen. The optical response of this assembly was observed to be linear, with a specified range of 1 pg mL^−1^ to 50 ng mL^−1^ with an improved detection limit of 0.084 pg mL^−1^.^171^ The electromechanical response of this sensor was satisfactory and comparable to traditional immunoassay methods.

Wei *et al.* designed a field effect-based semiconductor transistor-based high-performing biosensor to find PSA-based cancer symptoms. The authors fabricated the sensor assembly using CNTs with MoS_2_. The field effect mechanism is responsible for the rapid detection of target analyte due to heterostructure oxidation response on the selective electrodes. As per experimentation, the sensor has a linear response, which was varied from 10 to 13 μg μL^−1^ with a detection limit of 4 μg μL^−1^.^172^ The sensor has a higher stability approach for physiochemical temperaments.

### 3.6 Biosensors in cervical cancer detection

Anbu *et al.* developed a fluorescent-based sensor for detecting Cu^2+^ in relation to cervical cancer using naphthalimide–phenanthroimidazole. Under physiological conditions, the response of Cu^2+^ sustains paramagnetic ion reactions, due to which the response of this biosensor opens opportunities for fluorescent-based biosensor development. The response of this sensor was to initiate absorption through the stoichiometry process of transduction. The main benefit of this sensor was its economic factors. There was scope to improve the sensing mechanism to scale out the instrumental parameters for the target analyte.^173^

Pareek *et al.* designed a DNA-based biosensor to recognize Human Papilloma Virus-16 (HPV 16) biomarkers for early detection of cervical cancer. The sensing mechanism was based on an electrochemical phenomenon. The authors fabricated the biosensor using indium tin oxide, glass rod-based electrodes, and silver-coated AuNPs to enhance the sensing capability of the sensor. The sensor produced 0.54 mA aM^−1^ sensitivity with a tremendous linearity range of 100 aM to 1 μM. The detection limit of this assembly was 100 aM. This sensor also paves the way to early-stage DNA-based cancer analyte estimation.^174^

Rawat *et al.* prepared an electrochemical-based Geno sensor-based biosensor to detect cervical cancer. The main target analyte was HPV 16. The biosensor was fabricated using novel perylene tetracarboxylic acid with processed copper and graphene oxides to detect the target analyte. The sensor performed very well with a detection limit of 0.06 μA μM^−1^ mm^−2^. From the PoC system design, the Geno sensors set the benchmark regarding sensitivity and selectivity.^175^

## 4. Biosensors: modelling and measurements

Cancer is one of the most common life-threatening diseases, with the most remarkable fatality rate worldwide. Early cancer diagnosis is crucial for effective and successful therapy. Traditional cancer screening diagnostic procedures are expensive, time-consuming, and unsuitable for recurrent tests. However, biomarker-based cancer diagnosis is emerging as one of the most promising approaches for early detection, disease progression monitoring, and eventual cancer treatment. Parihar *et al.* developed a highly sensitive, selective, and specific electrochemical aptasensor called CeO_2_–GO/Apt/BSA/GCE for detecting EGFR biomarkers in PBS and actual saliva, sweat, and serum samples. The sol–gel process was used to manufacture the CeO_2_–GO nanocomposite. The oxygen atoms (O-atoms) on the faces and edges of CeO_2_ and GO enable considerable and facile EGFR protein immobilization/binding. The functionalization of CeO_2_ improves electroconductivity and inhibits aggregation in the CeO_2_–GO nanocomposite. The electrochemical characterization data indicate that the CeO_2_–GO nanocomposite has good electrochemical properties and a large surface area, making it suitable for the development of an electrochemical aptasensor with an ultra-low limit of detection (LOD) and a wide detection range for EGFR, ranging from 25.0 fg mL^−1^ to 25.0 ng mL^−1^. The manufactured aptasensor demonstrated outstanding results in genuine samples such as saliva, sweat, and serum. Therefore, it may be employed in clinical settings for non-invasive detection of cancer biomarkers.^176^

The synthesized material was utilized to create an electrochemical aptasensor capable of detecting epidermal growth factor receptor (EGFR) antigen. The developed sensor (Y_2_O_3_-rGO/Apt/BSA) has a broad linear detection range (10 fg mL^−1^ to 100 ng mL^−1^), a low detection limit of 0.251 fg mL^−1^, and an exceptional sensitivity of 51.96 μA fM^−1^ cm^−2^. The aptasensor also offers a simple and rapid way to detect EGFR antigen in biological serum samples. This work's simple and straightforward synthesis approach resulted in the manufacture of a high-purity, water-dispersible yttrium functionalized reduced graphene oxide nanocomposite for cost-effective, quicker, and ultrasensitive detection of breast cancer biomarkers utilizing electrochemical aptasensors.^177^

MXene-enabled electrochemical aptasensors have shown considerable potential in detecting cancer biomarkers at a femtomolar level. Furthermore, the stability, simplicity of synthesis, strong repeatability, and high specificity afforded by MXene-enabled aptasensors show potential for becoming the dominant diagnostic technique.^178^ The combination of carbon-based nanomaterials with highly specific aptamers (antibody mimics) can reduce sensor production costs while simultaneously improving effectiveness, precision, selectivity, and detection speed for a wide range of analytes. Furthermore, the integration of ideas and technology increases speedy design and smooth operations, therefore playing a vital role in numerous fields such as medical and healthcare services.^179^

Luo *et al.* demonstrated a locked nucleic acid (LNA) aided strand displacement reaction-based ratiometric electrochemical biosensor with greater repeatability to detect exosomal miR-21 originating from cancer with an LOD of 2.3 fM.^180^ Human epidermal growth factor receptor 2 (+) BT 474 (HER-2 (+) BT 474), a well-known BC biomarker, was easily identified from the first collected population using its complementary HER-2 antibody. The detection limit was as low as 4.7 × 10^5^ exosomes per μL.^181^

Canbaz and co-workers covalently attached an anti-HER-3 antibody to the modified Au electrode to detect the cancer-specific biomarker HER-3.^182^ Voltammetry and EIS were used to characterize the stated sensors, resulting in a linear detection range of 0.2–1.4 pg mL^−1^. Another group used a disposable electrochemical biosensor to detect the breast cancer-specific biomarker HER-2-ECD in human serum in the early stages, with a detection range of 10–150 ng mL^−1^ and a detection limit of 2.1 ng mL^−1^. The disclosed sensor's validity was verified by screening several human proteins and other cancer biomarkers (CA15-3).^183^

Heidari *et al.* described a GCE-based sensor that detects and quantifies p53 cancer biomarkers.^184^ This sandwich assay used GCE/CdS/p53-Ab1 and p53-Ab2-tGO-AuNPs to identify p53 cancer biomarkers. Epithelial ovarian cancer possesses a unique biomarker, human epididymis protein 4 (HE4), which can be detected in human serum down to 2.8 pM.^185^

Pacheco *et al.* demonstrated an Au-SPE-based biosensor to diagnose carbohydrate antigen 15-3 (CA 15-3) in human serum, which can be detected at concentrations as low as 1.5 U mL^−1^.^186^ Prasad *et al.* have developed a low-cost disposable paper-based electrode modified with a GO-based electrochemical biosensor to identify pancreatic cancer-specific biomarkers pseudopodium-enriched atypical kinase 1, SGK269 (PEAK1).^187^ The immunosensor operated well with a linear range of 10 –10^6^ pg mL^−1^ and a limit of detection of 10 pg mL^−1^. Muñoz-San Martín *et al.* recently announced an MBs-based microfluidic electrochemical biosensor capable of detecting tumoral hypoxia biomarker-hypoxia-inducible factor-1 alpha (HIF-1α) as low as 76 pg mL^−1^.^188^

## 5. Marketed biosensors

The biosensors are the future devices to detect the symptoms and progress in disorder for any illness such as cancer or other deadly disease in living beings. Various immunoassay-based biosensors are available on the market. The ORIGEN 1.5, M-series M-384 Analyzer, M-1 Analyzer, Luminex 200™ System, Sector PR2 Reader, Elecsys 1010 and 2010, as well as Modular analytics E-170 Analyzer, are the commercially available biosensor modules for measurement of cancer cells.^189,190^

As per the current scenario, the market of biosensors is worth more than 18 million worldwide due to an increment in reported cases. Adopting biocompatible materials, fabrication techniques, innovative design, and integration techniques support the commercialization of cancer-related biosensors. The recent advances in PoC technology demand sophisticated instruments to be involved in commercializing biosensors. The biosensing research and development filed under trials and needs the approval of governing bodies such as the FDA. The research community claims a linear response of biosensors with an expanded detection limit. However, the trials of these sensors are still underway to ensure stability concerning certain physio-environmental aspects. The Luminex 200™ System is recognized as commercially viable due to its 100 times reusability.^190^

For commercialization, there are certain aspects on which the research community must focus, such as stability, reusability, biocompatibility, cost of trial, and approval of ethical committees and governing bodies such as the FDA. With the advancement in IoT and NEMS-based technology, the search community must focus on developing commercial wireless biosensing modules to support cutting-edge technologies.^191^

## 6. Limitations of biosensor

Although the biosensors are designed and tested for the identification of target analyte with improvement in various electromechanical instrumentation factors, various aspects need to be considered as per PoC technology. The biosensor design must be user-friendly and have an ace of embodiment with testing modules.^192^ There is a need to fabricate more sophisticated and flexible electrode assemblies, embed low exciters in the transduction modules to improve the sensitivity of biosensors, enhance the effort in designing a photo detection-based system on a chip module to support non-invasive electronic portable devices, incorporate new amplification techniques and improve optical response. The biosensors lack stability under trials due to multilateral factors and only focus on one biomarker at a time, so false observations can be made, so multiplex detection is desired in the transduction process to improve the accuracy of biosensor results.^193–200^ The research community must focus on the fusion of artificial intelligence (AI) and machine learning (ML)-based algorithms to improve the performance of a biosensing mechanism in terms of stability and producible scalability. The biosensor field is an emerging area, and various aspects need to be incorporated as per the approval of medical and governing bodies for commercialization on a global scale.^201^

The primary obstacle in the development of transportable electrochemical paper-based biosensors is ensuring their dependability and capacity to identify many cytokines or other biomarkers concurrently in intricate biological specimens. Achieving the electrochemical transduction linked to hybridization events that do not genuinely need charge transfer processes is a difficulty in the development of electrochemical biosensors for nucleic acid BC indicators. The CNTs, graphene, graphene oxide (GO), reduced graphene oxide (rGO), graphene quantum dots (GQD), metal nanoparticles (MNPs) and nanostructures, magnetic beads, polymers, hydrogels, dendrimers, and nanocomposites are examples of nanostructured materials that are expected to be used more in the future and are becoming more and more important in their fabrication.^202^

When applied to actual samples, graphene-based biosensors are unable to produce the intended detection outcomes. This is primarily due to the non-specific nature of the interfacial interactions between graphene and different chemical and biological substances toward the intended targets. Furthermore, biological sample preparation—which may entail laborious and time-consuming steps like separation or preconcentration—is required prior to the completion of the tests. However, it can be difficult to disperse graphene nanoparticles in other materials, and doing so necessitates functionalizing the graphene surface, which raises the cost of producing biosensors. The identification of precise and sensitive biomarkers can significantly enhance cancer diagnosis. Despite this, not much progress has been made in the creation of extremely sensitive and repeatable biosensors for early cancer diagnosis or in the identification of novel biomarkers. Despite several publications, it might be challenging to create substrates that are highly specific to a particular cancer biomarker because most biomarkers are often present in multiple malignancies. Therefore, another significant difficulty for electrochemical biosensors is devising ways to detect many biomarkers while avoiding false positives concurrently. It is also probable that cancer biosensors do not cover the complete spectrum of biomarker concentrations due to the low concentration of the majority of cancer biomarkers.^203,204^

The main disadvantages of aptasensors that hinder the broader application of aptamer DNAzyme conjugate as therapeutics are noted as their toxicity, limited metabolism, and nuclease degradation in a physiological environment. There is still a long way to go before DNAzyme-assisted aptasensors can be used to identify cancer biomarkers *in vivo* and in complex clinical specimens. Since the majority of human malignancies contain several biomarkers, it is still difficult to detect specific biomarkers since developed DNAzyme aptasensors interfere with the variety of biomarkers present in cancer cells. Moreover, it is difficult to measure the local amounts of signals that cancer cells emit *in vivo*.^205^ The development of 3D-printed electrochemical sensors for cancer protein biomarkers often requires the printing of many components, which is hampered by the significant variance in inter-material adhesion forces and the highly restricted printing processes capable of attaining such a characteristic. Furthermore, standard 3D printing materials and procedures are not very compatible with biomolecules, especially when it comes to the high energy needed for printing processes and the high-intensity laser used in stereolithographic 3D printing, which makes it impossible to print these biomolecules directly.^206^

## 7. Clinical trials

**Fig. 4:**
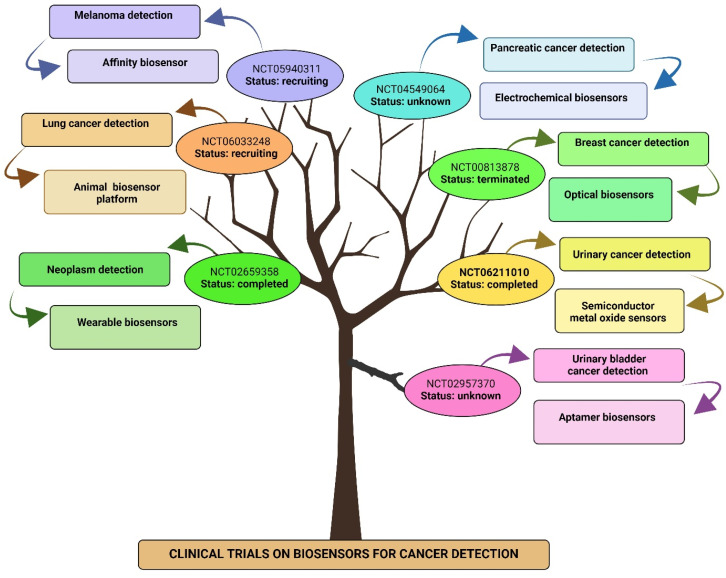
Schematic representation of clinical trials on biosensors for cancer detection.

Fig. 4 schematically represents the clinical trials on biosensors for detecting various cancers.^207–213^

## 8. Conclusion and future aspects

The difficulties and advancements in the development of biosensors have made it easier to analyze cancer biomarkers. Without a doubt, developments in biosensor technology will contribute to the development of medical diagnostic equipment for use at the bedside. This review provides a quick overview of many popular sensor types, including electrochemical, optical, and piezoelectric sensors, that are often employed to identify common tumor markers. The electrochemical sensors are capable of identifying common tumor markers among them. A range of novel materials, including chelated Eu^3+^ materials and GO NPs, have also been developed with the specific goal of detecting the stomach cancer marker CEA. These materials provide excellent sensitivity, good selectivity, and quick analytical speeds. Early diagnosis, better patient outcomes from therapy, and enhanced quality of life are all aided by it. Moreover, optical biosensors are crucial for drug development, clinical diagnostics, and other areas. Optical biosensors offer excellent stability, great dependability, and high sensitivity. They have more significant anti-interference capabilities and greater accuracy when compared to standard electrochemical biosensors. Furthermore, adding protamine to plasma can increase the measured LOD value in the experiment employing a piezoelectric biosensor to detect PSA because protamine can bind to fibrinogen.

The use of biosensors for early clinical diagnosis and prognostic monitoring in real-world settings remains challenging. First, because biomarkers are exceedingly intricate in living things, their detection does not imply the presence of cancer; genetic and environmental factors must also be considered. Second, it is difficult to distinguish the kind of cancer alone by looking for biomarkers, which is harmful to early clinical diagnosis and therapy due to gene crossing in cancer cells. Finally, taking optical sensors as an example, the bulk of those now on the market are still difficult. As a result, further work is needed to improve the biosensors' stability, reusability, and compatibility with biological fluids. In the future, they should be more compact, integrated, and mobile. If these issues are addressed, biosensors can be used in clinical laboratories and hospitals to diagnose and treat cancer at a low cost.

## Data availability

No new data was generated during the preparation of this manuscript.

## Author contributions

Conceptualization, S. G., V. M., and Y. M.; writing—original draft preparation, S. G., V. M., A. A. A. A., A. A., R. K., and Y. M.; writing—review and editing, V. M., A. A. A. A., A. A., M. E. T., and Y. M.; supervision, V. M., and Y. M. All authors have read and agreed to the published version of the manuscript.

## Conflicts of interest

The authors declare no conflict of interest.
